# Emotion Regulation Deficits in Adolescent Girls with Major Depression, Anorexia Nervosa and Comorbid Major Depression and Anorexia Nervosa

**DOI:** 10.1007/s10578-022-01353-4

**Published:** 2022-04-12

**Authors:** Carolin Zsigo, Anca Sfärlea, Carolin Lingl, Charlotte Piechaczek, Gerd Schulte-Körne, Lisa Feldmann, Ellen Greimel

**Affiliations:** Department of Child and Adolescent Psychiatry, Psychosomatics and Psychotherapy, Hospital of the Ludwig-Maximilians-University (LMU) Munich, Pettenkoferstr. 8a, 80336 Munich, Germany

**Keywords:** Major depression, Anorexia nervosa, Emotion regulation, Comorbidity, Adolescence

## Abstract

In adolescence, both major depression (MD) and anorexia nervosa (AN) are associated with deficits in emotion regulation (ER). However, studies have yet to compare ER profiles within the disorders and the effect of comorbid MD+AN is unclear. This study examined the habitual use of ER in 229 girls, aged 12–18 years, with MD (n = 84), AN (n = 37), comorbid MD+AN (n = 25), and healthy girls (n = 83). Girls with MD, AN and MD+AN reported more maladaptive and less adaptive ER strategies than healthy girls. MD and MD+AN groups showed more frequent use of maladaptive ER compared to only AN, with no differences between only MD and MD+AN. This suggests that MD+AN is not necessarily associated with higher ER deficits, rather, an additional diagnosis of MD exacerbates impairments in AN. Identifying specific ER profiles can provide important targets in prevention and treatment for AN, MD and AN+MD.

**Supplementary Information:**

The online version contains supplementary material available at 10.1007/s10578-022-01353-4.

## Introduction

During adolescence, young people undergo crucial milestones in their physical, psychological and social development while at the same time facing an increasing risk for the onset of psychopathological disorders [1]. Both major depression (MD) and anorexia nervosa (AN) increase in prevalence during adolescence: the risk of suffering from a depressive episode rises to a 12-month prevalence rate of about 7.5% [2], while a 12-month prevalence of about 0.5% has been reported for a diagnosis of AN [3, 4]. Both disorders are more frequent in adolescent girls than in boys [2–4] and comorbidity between the two is high. About 30–40% of adolescents with AN are diagnosed with comorbid MD [3, 5], with some reports documenting even higher numbers (for a review, see [6]). On the flip side, about 4% of adolescents with MD have been found to present a comorbid eating disorder [7]. The presence of MD or depressive symptoms in AN is related to higher AN symptomatology [8–10], as well as a poorer outcome [11] and poorer quality of life [12] in affected patients.

One characteristic of both disorders is a deficit in emotion regulation [13]. Emotion regulation (ER) describes the processes and strategies individuals make use of to influence their emotions, as well as their experience and expression of these emotions [14].

In the classification of ER strategies, the terms adaptive and maladaptive ER strategies have been established [15, 17, 18]. Adaptive strategies comprise functional ER strategies such as reappraisal or distraction that enable coping with difficult situations [17, 19]. They are positively associated with psychological resilience [20] and subjective well-being [16]. Maladaptive strategies comprise dysfunctional strategies such as aggressive actions [21, 22], avoidance [23, 24] and rumination [25]. Maladaptive strategies are regarded as risk factors for the development of psychopathology (for a meta-analysis, see [17]). The detrimental effect of maladaptive ER strategies on psychopathology seems to be stronger than the protective effect of adaptive ER strategies [19, 26].

Research suggests that disturbances in ER during adolescence are specifically related to the development and maintenance of MD [27, 28]. Youth with depression exhibit a higher use of maladaptive strategies [17, 29]. Of these, especially rumination has been strongly associated with the disorder and has been found to predict the onset of future depressive episodes in both adolescents and adults [25, 30]. Strategies of avoidance and suppression have also been connected to depressive psychopathology in adolescence (for a meta-analysis, see [31]). In healthy adolescents, it has additionally been found that more maladaptive ER strategy use was associated with more depressive symptoms [32]. Research looking at adaptive ER strategies in adolescents with MD is sparse. In adults with MD, a reduced use of adaptive strategies, including reappraisal, acceptance, and distraction, has been observed [33]. A more frequent use of adaptive strategies in adults with MD has also been associated with fewer depressive symptoms [29, 34, 35]. In healthy adolescents, depressive symptomatology was connected to a less frequent use of reappraisal, problem solving and acceptance ([29], for a meta-analysis, see [31]).

There is less research on ER in AN than in MD, especially among adolescents. Two studies showed that adolescent girls with AN displayed more difficulties in ER compared to a typically developing group [36, 37]. AN in adolescence is also connected to less acceptance [37, 38] and eating disorders in general have been connected to more emotional suppression and less cognitive reappraisal in youth [39]. There are more findings in adults with AN, with studies similarly showing that they use less acceptance than healthy controls [13, 40]. Results of a meta-analysis further showed that adults with AN regulate their emotions by avoiding conflict, suppressing their emotions and ruminating [41]. These deficits in adaptive ER have also been shown to be related to both higher eating disorder severity and a prolonged course of AN in adults [42]. In adults with AN, theoretical concepts underlying radically open dialectical behavior therapy (RO DBT) describe AN as an overcontrolled disorder, in which the problem lies less in emotional dysregulation but rather an overabundance of emotional control. As such, the disorder is characterized by inhibited emotional expressiveness, rigidity, and impaired emotion recognition [43, 44]. This can then manifest in maladaptive behavioral tendencies similar to those associated with emotion dysregulation.

To our knowledge, there is little research directly comparing ER deficits between adolescents with AN and adolescents with MD. Important first insights come from Nalbant et al. [36], who found that in adolescent girls with AN, depressive symptoms were positively correlated with difficulties in ER, suggesting that the presence of depressive symptomatology might enhance deficits of ER in AN. However, this study only assessed depressive symptoms, without assessing a possible diagnosis of MD, and the relationship between a comorbid diagnosis of AN and MD and ER thus remains unknown. Additionally, the study did not report on whether eating disorder severity is correlated with ER. No study to date has differentiated between ER profiles of AN patients with and without MD and compared adolescents with AN or MD to adolescents suffering from both disorders.

However, it is highly important to identify specific profiles of ER strengths and deficits in both AN and MD in order to develop new and optimize existing interventions targeting ER. Insight into the question on how the comorbidity of AN and MD is related to ER is equally relevant in the light of the high prevalence and comorbidity of the two disorders. Additionally, such research could also be beneficial for at risk populations. ER has been reported to be a precursor for symptoms of MD [27, 45], so identifying and addressing specific ER deficits could help develop preventative strategies. Finally, ER profiles of AN and MD can also inform theoretical models of the two disorders.

The aim of the present study was therefore to identify impairments in maladaptive and adaptive ER strategies in adolescent patients with MD, AN and in adolescents diagnosed with both disorders compared to healthy youths. Based on previous findings, we expected a more frequent use of maladaptive ER strategies in adolescents with MD compared to healthy adolescents, specifically higher use of rumination and withdrawal. Moreover, we hypothesized a less frequent use of adaptive ER strategies, specifically lower problem solving, acceptance, and reappraisal [17, 31, 33]. In adolescents with AN, compared to healthy adolescents, we similarly expected a more frequent use of maladaptive strategies and a less frequent use of adaptive ones [36, 37]. Based on findings in adults with AN, we further specifically expected a higher use of the maladaptive strategies withdrawal and rumination alongside a lower use of the adaptive strategy acceptance [13, 40].

With regards to the lack of literature on comorbid AN and MD with respect to ER, we posed no specific hypotheses for the exact ER profile in this group. Based on findings that a comorbid presentation of AN and MD can forecast a poorer outcome [8, 11, 12], however, we expected that the *extent* of the reported deficits will be exacerbated when a comorbid diagnosis is present.

Lastly, we expected that the severity of psychopathological symptoms is correlated with the severity of deficits in adaptive and maladaptive ER [32, 35, 46]. In more detail, we expected that higher symptomatology of depression or anorexia is associated with more frequent use of maladaptive strategies and less frequent use of adaptive ones. Based on previous findings [17, 19], we also expected that the connection of psychopathological symptoms with maladaptive ER strategies will be stronger than the one with adaptive ones.

## Methods

### Study Population

The study sample consisted of 229 adolescent girls with a mean age of 15.39 years. Of these, 84 were diagnosed with major depression (MD-Only), 37 were diagnosed with anorexia nervosa (AN-Only), 25 were diagnosed with both anorexia nervosa and major depression (MD+AN) and another 83 made up the healthy control group (HC), who never met any criteria of current or past psychiatric disorders. To be included in the respective clinical group, criteria for current MD and/or AN had to be met according to the classification specified in the ICD-10 [47]. The groups did not differ significantly in age or IQ (*p*_s_ > 0.05), as can be seen alongside characteristics of the four groups in Table 1.

**Table 1:** Sample characteristics and descriptive data

	HC (n = 83)	MD-only (n = 84)	AN-only (n = 37)	MD + AN (n = 25)	ANOVA		Post-hoc tests
	*M* (*SD)*	*M* (*SD)*	*M* (*SD)*	*M* (*SD)*	*F*_*3,225*_	*p*	
Age	15.55 (1.75)	15.12 (1.49)	15.54 (1.64)	15.49 (1.74)	1.16	n.s	–
IQ	108.92 (11.68)	109.34 (11.14)	108.50 (12.23)	104.32 (15.44)	1.17	n.s	–
BDI-II	2.93 (3.13)	31.00 (11.67)	18.38 (8.36)	35.40 (10.98)	237.04^a^	< .001	HC < AN < MD, MD + AN
EDI-2	–	–	284.27 (50.74)	336.87 (47.16)	–	–	AN < MD + AN^b^
Drive for Thinness	–	–	27.82 (9.69)	33.52 (8.55)	–	–	AN < MD + AN^c^
Bulimic Symptoms	–	–	11.86 (5.00)	12.78 (6.49)	–	–	AN = MD + AN^d^
Body Dissatisfaction	–	–	37.85 (9.92)	44.65 (9.16)	–	–	AN < MD + AN^e^

*HC* healthy control; *MD* major depression; *AN* anorexia nervosa; *MD*+*AN* major depression and anorexia nervosa; *ER* Emotion Regulation; *M* mean; *SD* standard deviation; *BDI* Beck’s Depression Inventory; *EDI* Eating Disorder Inventory. For BDI-II and EDI-2, raw scores are reported

^a^Due to heterogeneity in variances, a Welch ANOVA was calculated with *F*(3, 66.12) = 237.04

^b^A t-test was conducted with *t*(58) = 4.01, *p* < .01

^c^A t-test was conducted with *t*(58) = 2.31, *p* = .024

^d^A t-test was conducted with *t*(58) = 0.66, *p* > .05

^e^A t-test was conducted with *t*(58) = 2.66, *p* = .010

Data were collected within the framework of larger projects on emotion processing and ER in AN and MD, respectively, at the Department of Child and Adolescent Psychiatry, Psychosomatics and Psychotherapy at the Hospital of the Ludwig-Maximilians-University Munich. Participants in the healthy control group were recruited from the community, while participants in the three clinical groups (AN-Only, MD-Only and comorbid MD+AN) were inpatients or outpatients from the Department of Child and Adolescent Psychiatry. All procedures were approved by the local ethics committee. All participants were informed in detail about the procedures and the aims of the study and provided written informed assent. Additionally, written informed consent was obtained from at least one legal guardian, after the legal guardian(s) had been informed about all aspects of the study. In return for their participation, all participants received vouchers.

Only girls between 12 and 18 years old who reached an IQ ≥ 80 were included in the study. Comorbidities were investigated in all three clinical groups. Overall, 164 participants (71.6%) did not show any comorbid disorders to MD and/or AN. 33 (14.3%) had one additional disorder, 18 (7.9%) showed two and 14 (6.2%) had three or more comorbid diagnoses. Anxiety disorders were the most common in every group, with 43 participants in the MD-Only group (51%) having an additional anxiety disorder, 5 in the AN-Only group (14%) and 11 in the MD+AN group (44%). Comparable numbers have been reported, with about 25–50% of adolescents with MD [48, 49] and about 10–25% of adolescents with AN [50] typically presenting a comorbid anxiety disorder.

### Measures

Psychiatric diagnoses were assessed in all participants using a semi-structured clinical interview (Kinder-DIPS; [51]). The Kinder-DIPS is a well-established German interview for the diagnosis of a wide range of axis I psychiatric disorders, including e.g. major depression, anxiety and eating disorders, in children from ages 6 to 18. The interview shows high retest and inter-rater reliabilities and has been reported to have good validity [51, 52]. All interviews were conducted by psychology students who were instructed and supervised by licensed psychologists who had completed a certified training course by an author of the Kinder-DIPS [51].

IQ was assessed with the short version of the CFT-20-R (Grundintelligenztest Skala 2-Revision; [53]). In prior research, the short version of the CFT 20-R showed sufficient retest reliability (r = 0.85) and construct validity [53]. In few inpatients, comparable measures, like the WISC-IV [54] or WAIS-IV [55], were available from routine testing and used instead. The IQ score was calculated by age-specific standardized values [53].

In all participants depressive symptoms were assessed with the German version of the BDI-II (Beck’s Depression Inventory II), a self-report questionnaire with 21 items that allows for a valid assessment of depressive symptomatology in adolescents [56]. The following cut-off values indicate depression severity: 0–8 = no depression; 9–13 = minimal depression; 14–19 = mild depression, 20–28 = moderate depression; 29–63 = severe depression [56]. In our sample, internal consistency was excellent (Cronbach’s α = 0.96).

In patients with AN, eating disorder symptomatology was assessed with the German long version of the EDI-2 (Eating Disorder Inventory 2), which consists of 91 items and allows for a valid assessment of disordered eating [57]. Items are grouped in eleven different scales (drive for thinness, bulimic symptoms, body dissatisfaction, ineffectiveness, perfectionism, interpersonal distrust, interoceptive awareness, maturity fears, asceticism, impulse regulation and social insecurity). Internal consistency in our sample was excellent (Cronbach’s α = 0.97).

For the assessment of ER strategies, we used the FEEL-KJ (Fragebogen zur Erhebung der Emotionsregulation bei Kindern und Jugendlichen; [16]), a self-report questionnaire with 90 items which assesses both cognitive- and behavioral ER strategies in children and adolescents. It consists of seven adaptive (acceptance, cognitive problem solving, problem-oriented action, positive mood enhancement, revaluation, forgetting, distraction) and five maladaptive (giving up, aggression, withdrawal, self-devaluation and rumination) strategies. While there is no scale measuring reappraisal, in which we expected adolescents with MD to show deficits, the scale “revaluation” is conceptually similar, as both focus on changing the emotions associated with a certain situation by changing one’s thoughts and judgment about it. Thus, we assumed that possible deficits in reappraisal would also be apparent in the scale “revaluation”.

Each strategy is assessed by two items which are repeated in the context of three different emotions (anxiety, sadness and anger), e.g. “When I am angry, I think about things that make me happy”, “When I am sad, I keep my feelings to myself”. Detailed descriptions and example items for each primary strategy can be found in Supplementary Table 1. Each item is rated on a five-point Likert scale according to how often the strategy is applied in daily life. The sum of all items from the seven primary adaptive strategies forms the composite scale “total adaptive ER strategies”, while the sum of all items from the five maladaptive strategies forms the composite scale “total maladaptive ER strategies”. These scales have also been referred to as “secondary scales” (e.g. in the FEEL-KJ manual, [16]).

Internal consistencies in our sample were excellent for total adaptive (Cronbach’s α = 0.96) and total maladaptive strategies (Cronbach’s α = 0.92) and ranged from α = 0.73 (rumination) to α = 0.93 (distraction) in primary scales. Data analysis of the FEEL-KJ, as described below, was based on raw scores. For descriptive purposes, T-values were calculated for total adaptive and maladaptive and primary FEEL-KJ scales using the standard values as provided by the FEEL-KJ manual [16]. The results can be found in Supplementary Table 2. Consistencies for all measures and scales can be found in Supplementary Table 3.

### Data Analysis

Statistical analyses were conducted with IBM SPSS Statistics 26. To investigate differences in depressive symptoms, we employed a Welch’s ANOVA due to heterogeneity of variance. The AN-Only and MD+AN groups were further compared in eating disorder symptomatology based on independent t-tests. We compared differences in both the total score of the EDI-2, as well as in each of the three scales “drive for thinness”, “body dissatisfaction”, and “bulimic symptoms”, which are commonly applied to assess specifically disordered eating behavior and cognitions (e.g. [58]).

To investigate the associations between MD, AN and their comorbid diagnosis and the habitual use of total adaptive and maladaptive ER strategies, a MANOVA with the factors group (MD-Only, AN-Only, MD+AN, HC) and total ER strategies (adaptive, maladaptive) was conducted. In a second step, to investigate the associations between the groups and the habitual use of primary adaptive ER strategies, we conducted a MANOVA with the factors group (MD-Only, AN-Only, MD+AN, HC) and primary adaptive ER strategies (acceptance, cognitive problem solving, problem-oriented action, positive mood enhancement, revaluation, forgetting, distraction). The same procedure was repeated for maladaptive ER strategies, employing a MANOVA with the factors group (MD-Only, AN-Only, MD+AN, HC) and primary maladaptive ER strategies (giving up, aggression, withdrawal, self-devaluation, rumination). All significant results were followed up with univariate analyses of variance (ANOVAs), of which significant outcomes were further investigated with post-hoc comparisons. If group variances were homogeneous, Tukey–Kramer post-hoc comparisons were made. The Tukey–Kramer method is recommended as it is less sensitive to Type-I errors than comparable measures when group sizes are unequal [59, 60]. If variances were heterogeneous, the Games-Howell procedure was employed as an improved version of the Tukey–Kramer method for unequal variances, which is able to maintain the set significance level even when sample sizes are different [61]. Due to the tests’ abilities to maintain the set significance level under multiple tests and different sample sizes, we did not further control for multiple testing.

To assess whether the use of adaptive or maladaptive ER strategies was correlated with symptom severity, bivariate Pearson correlation analyses were performed in all four groups for total adaptive and maladaptive ER strategies and depressive symptoms, as measured by the BDI-II. For the AN-Only and MD+AN group, bivariate correlation analyses were performed to assess the relationship between total adaptive and maladaptive ER strategies and eating disorder symptoms, measured by the EDI-2. We correlated both the total score of the EDI-2, as well as the each of the three scales “drive for thinness”, “body dissatisfaction”, and “bulimic symptoms” with total adaptive and maladaptive ER scales, respectively. For multiple correlations, all relevant p-values were controlled via the Bonferroni-Holm procedure.

## Results

### Sample Characteristics

The groups showed significant differences in depressive symptomatology (*F*(3, 66.13) = 237.04, *p* < 0.001). As can be expected, post-hoc Games-Howell tests revealed that the HC group showed significantly less depressive symptoms than the three clinical groups (*p*_s_ < 0.001). Further, it was confirmed that both MD-Only and MD+AN groups showed higher depressive symptomatology than the AN-Only group (*p*_s_ < 0.001), while MD-Only and MD+AN groups did not differ (*p* > 0.05). For eating disorder symptomatology, the MD+AN group showed significantly higher eating disorder symptoms than the AN-Only group, both for EDI-2 total (*t*(58) = 4.01, *p* < 0.001) as well as the EDI-2 scales “drive for thinness” (t(58) = 2.31, *p* = 0.024) and “body dissatisfaction” (t(58) = 2.66, *p* = 0.010) but not “bulimic symptoms” (t(58) = 0.62, *p* > 0.05). Note that bulimic symptoms were generally low in our sample (see Table 1 for descriptive measures). Significant differences survived Bonferroni-Holm correction for multiple testing.

These differences between the two groups are likely due to inherent characteristics of the two groups, as it has been well documented that an additional diagnosis of MD and/or high depressive symptomatology in AN forecasts more difficulties and higher severity of illness [8, 11, 12]. For both depressive symptom and eating disorder severity, we assume these differences to be due to inherent group characteristics and thus, controlling for these differences between the groups would remove variance essential to the group differences we aim to investigate (see also [62]). We therefore decided not to control for differences in depressive or eating disorder symptomatology between the groups.

### Total Adaptive and Maladaptive ER Scales

**Fig. 1:**
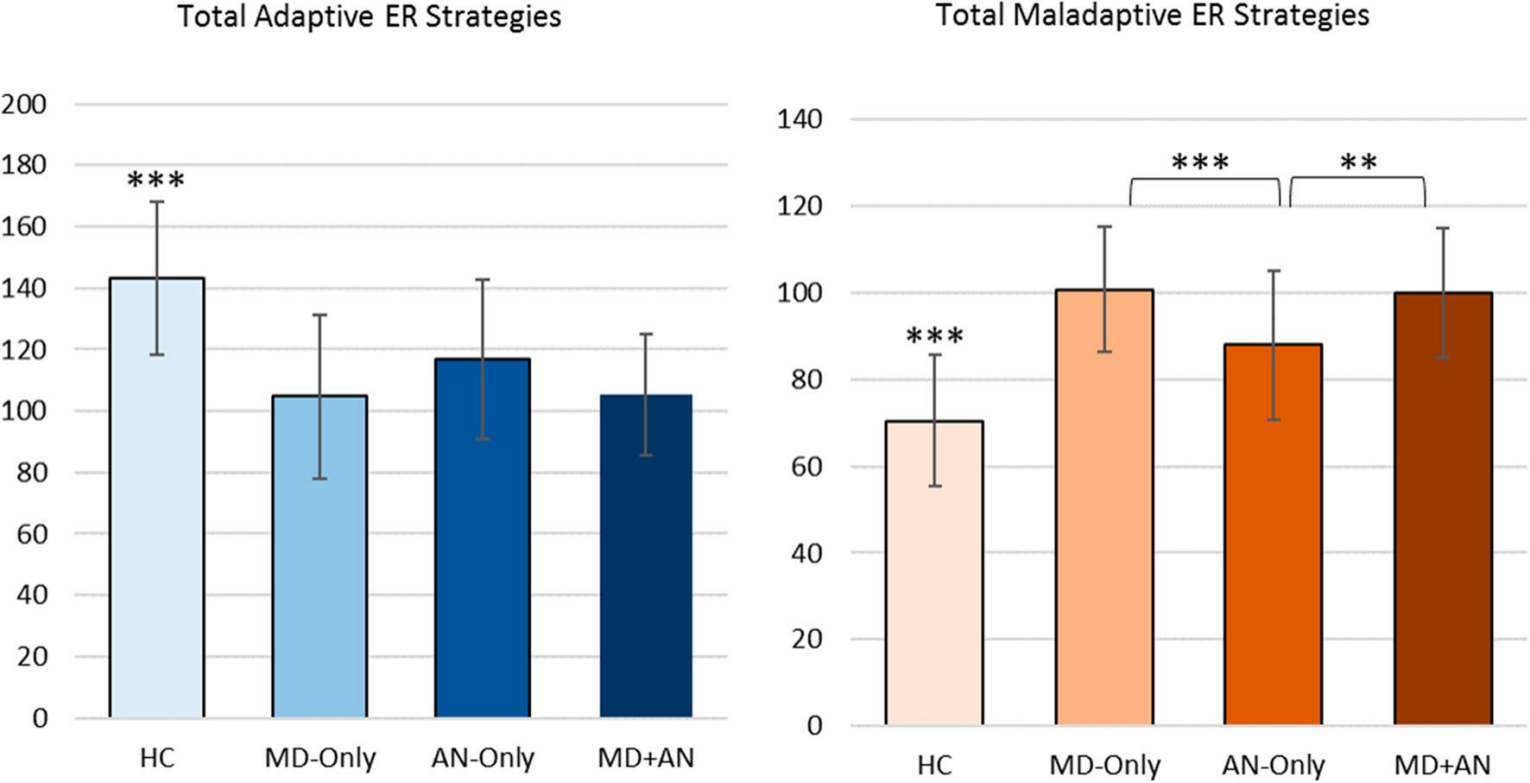
Group differences in total adaptive and maladaptive ER strategies. *p < .05, **p < .01, ***p < .001. Note: *HC* healthy control; *MD* major depression; *AN* anorexia nervosa; *MD + AN* major depression and anorexia nervosa. Error bars show standard deviations. The *y*-axis displays raw FEEL-KJ scores for the respective scales

The MANOVA investigating the associations between the factor group and total adaptive and maladaptive ER strategies was significant (*F*(6, 448) = 34.22, *p* < 0.001, partial η^2^ = 0.31, Wilk’s Λ = 0.47). Follow-up univariate ANOVAs revealed significant associations between the factor group and both total adaptive (*F*(3, 225) = 36.60, *p* < 0.001, partial η^2^ = 0.33) and maladaptive (*F*(3, 225) = 61.82, *p* < 0.001, partial η^2^ = 0.45) ER strategies. All group differences for total adaptive and maladaptive ER strategies can be found in Fig. 1.

For total adaptive ER strategies, Tukey–Kramer post hoc analyses showed that the three clinical groups reported significantly less adaptive ER strategies than the HC group, *p*_*s*_ < 0.001 (HC vs. MD-Only: *M*_Diff_ = 38.41, 95%-CI [28.31, 48.50]; HC vs. AN-Only: *M*_Diff_ = 26.40, 95%-CI [13.51, 39.30]; HC vs. MD+AN: *M*_Diff_ = 37.90, 95%-CI [23.02, 52.79]). There were no significant differences between the three clinical groups (*p*_s_ > 0.05).

For total maladaptive ER strategies, Tukey–Kramer post hoc analyses showed that the three clinical groups also reported significantly more maladaptive ER strategies than the HC group, *p*_*s*_ < 0.001 (HC vs. MD-Only: *M*_Diff_ = − 30.26, 95%-CI [− 36.35, − 24.19]; HC vs. AN-Only: *M*_Diff_ = − 17.51, 95%-CI [− 25.28, − 9.75]; HC vs. MD+AN: *M*_Diff_ = − 29.57, 95%-CI [− 25.28, − 20.60]). Furthermore, both MD-Only and MD+AN groups applied significantly more maladaptive strategies than AN-Only (MD vs. AN-Only: *p* < 0.001, *M*_Diff_ = 12.76, 95%-CI [5.00, 20.51]; MD+AN vs. AN-Only: *p* = 0.013, *M*_Diff_ = 12.05, 95%-CI [1.88, 22.23]). There were no differences between MD-Only and MD+AN (*p* > 0.05).

### Primary Adaptive ER Strategies

**Fig. 2:**
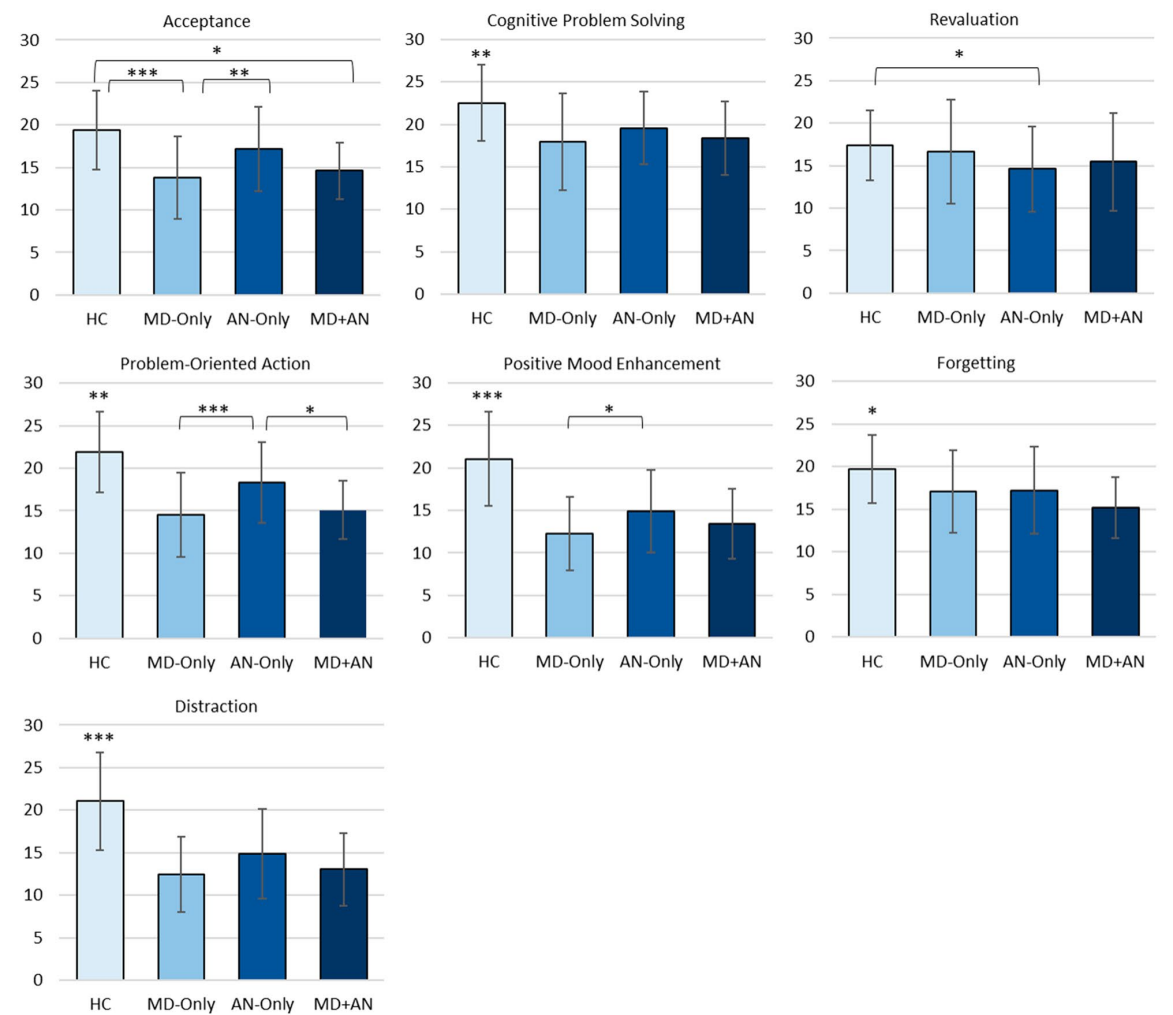
Group differences in primary adaptive ER strategies. *p < .05, **p < .01, ***p < .001. Note: *HC* healthy control; *MD* major depression; *AN* anorexia nervosa; *MD + AN* major depression and anorexia nervosa. Error bars show standard deviations. The *y*-axis displays raw FEEL-KJ scores for the respective strategies

The MANOVA investigating the associations between the factor group and primary adaptive ER strategies was significant (*F*(21, 629.4) = 8.35, *p* < 0.001, partial η^2^ = 0.21, Wilk’s Λ = 0.49). Follow-up univariate ANOVAs revealed further significant associations between the factor group and all primary adaptive strategies, with *p*_s_ < 0.001 for acceptance (*F*(3, 225) = 21.93, partial η^2^ = 0.23), cognitive problem solving (*F*(3, 225) = 13.32, partial η^2^ = 0.15), problem-oriented action (*F*(3, 225) = 37.98, partial η^2^ = 0.34), positive mood enhancement (*F*(3, 225) = 48.98, partial η^2^ = 0.40), forgetting (*F*(3, 225) = 9.12, partial η^2^ = 0.12) and distraction (*F*(3, 225) = 44.42, partial η^2^ = 0.37), and *p* = 0.049 for revaluation (*F*(3, 225) = 2.66, partial η^2^ = 0.03). Group differences for all primary adaptive ER strategies can be found in Fig. 2.

Post-hoc analyses revealed that for all strategies except acceptance and revaluation, the three clinical groups applied less of those strategies than the HC group (*p*_s_ < 0.05). For acceptance, the MD-Only group (*p* < 0.001) and the MD+AN group (*p* < 0.001) applied less of this strategy than the HC group, while there was no difference between the HC group and the AN-Only group (*p* = 0.070). With respect to revaluation, the AN-Only group reported less frequent habitual use of this strategy compared to the HC group (*p* = 0.025), with no further differences between other groups (*p*_s_ > 0.05).

Patients with MD-Only applied less acceptance (*p* = 0.002), problem-oriented action (*p* < 0.001) and positive mood enhancement (*p* = 0.034) than patients with AN-Only. The MD+AN group applied less problem-oriented action than AN-Only (*p* = 0.041) but otherwise showed no significant differences to AN-Only or MD-Only in their use of the primary strategies.

### Primary Maladaptive ER Strategies

**Fig. 3:**
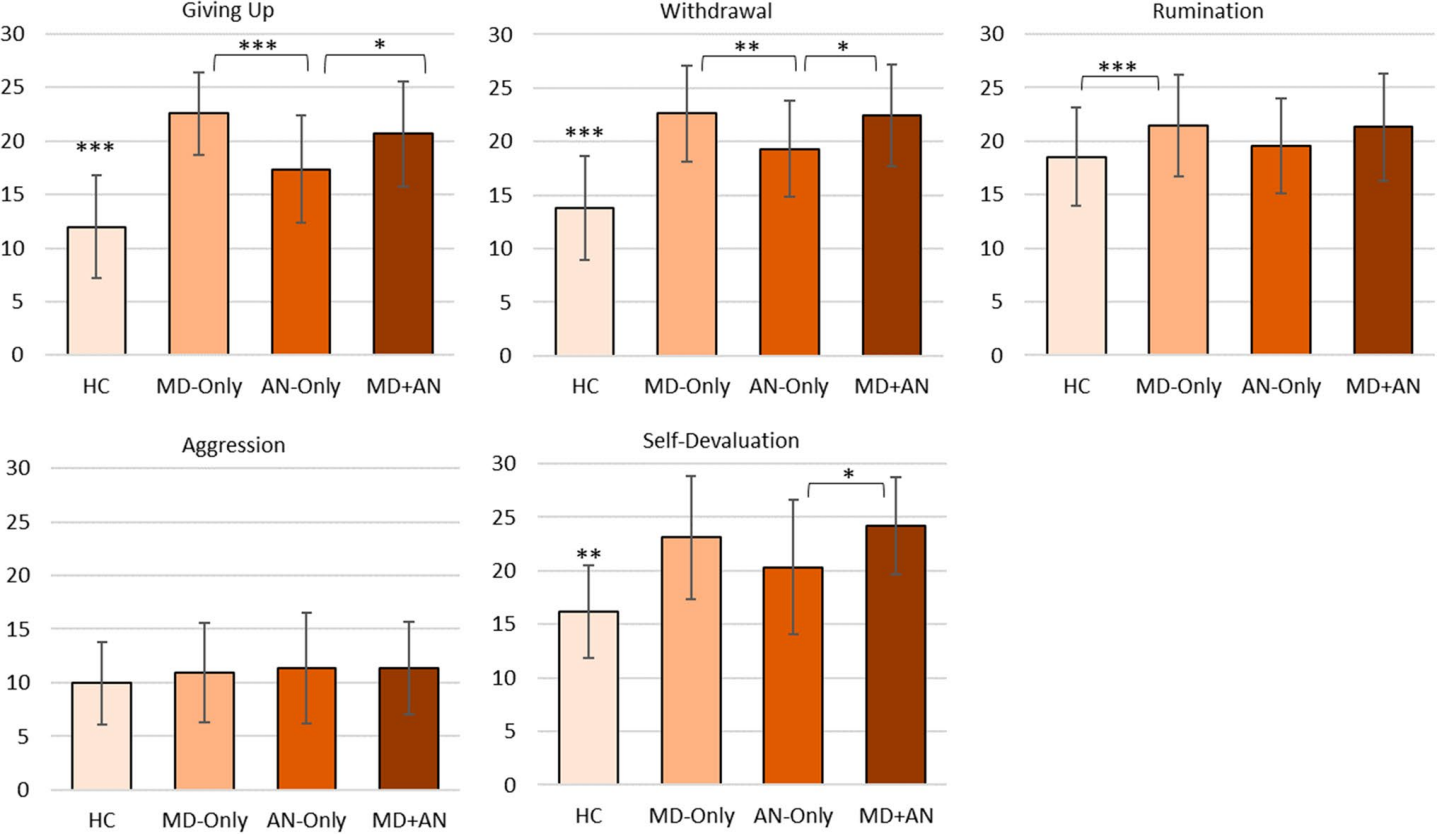
Group differences in primary maladaptive ER strategies. *p < .05, **p < .01, ***p < .001. Note: *HC* healthy control; *MD* major depression; *AN* anorexia nervosa; *MD + AN* major depression and anorexia nervosa. Error bars show standard deviations. The *y*-axis displays raw FEEL-KJ scores for the respective strategies

The MANOVA investigating the associations between the factor group and primary maladaptive ER strategies was significant (*F*(15, 610.49) = 15.21, *p* < 0.001, partial η^2^ = 0.25, Wilk’s Λ = 0.42). Follow-up univariate ANOVAs revealed significant associations between the factor group and the strategies giving up (*F*(3,225) = 79.19, *p* < 0.001, partial η^2^ = 0.51), withdrawal (*F*(3,225) = 57.60, *p* < 0.001, partial η^2^ = 0.43), self-devaluation (*F*(3,225) = 30.67, *p* < 0.001, partial η^2^ = 0.29) and rumination (*F*(3,225) = 6.14, *p* < 0.001, partial η^2^ = 0.07). There was no significant association between the factor group and the ER strategy aggression (*p* > 0.05). Group differences for all primary adaptive ER strategies can be found in Fig. 3.

Post-hoc analyses of the four significant strategies revealed that for giving up, withdrawal and self-devaluation, all three clinical groups applied significantly more of those strategies than the HC group (*p*_s_ < 0.01). For rumination, the MD-Only group applied more than the HC group (*p* < 0.001), with no difference between the AN-Only group and the HC group (*p* > 0.05) and the MD+AN group and the HC group (*p* > 0.05). Participants with MD-Only applied more giving up (*p* < 0.001) and withdrawal (*p* = 0.002) than those with AN-Only. The MD+AN group applied more giving up (*p* = 0.027), withdrawal (*p* = 0.048) and self-devaluation (*p* = 0.031) than the AN-Only group but showed otherwise no significant differences to the AN-Only or MD-Only groups (*p*_*s*_ > 0.05).

### Additional Covariate Analyses

In order to identify potential covariates, we correlated the variables age, IQ and age of onset with all twelve primary scales of the FEEL-KJ. After Bonferroni-Holm correction, only two correlations remained significant: age correlated with problem-oriented action (*r* (227) = 0.22, *R*^2^ = 0.05, *p* = 0.001) and with cognitive problem solving (*r* (227) = 0.24, *R*^2^ = 0.06, *p* < 0.001).

To control for age within these two scales, we conducted ANCOVAs with the factors group (HC, MD-Only, AN-Only, MD+AN) and the two strategies problem-oriented action and cognitive problem solving respectively, including age as a covariate. The ANCOVA for problem-oriented action revealed that the factor group was significant (*F*(3, 225) = 36.78, partial η^2^ = 0.33, *p* < 0.001), with age as a significant covariate (*p* = 0.003). The ANCOVA for cognitive problem solving revealed that the factor group was significant (*F*(3, 225) = 12.46, partial η^2^ = 0.14, *p* < 0.001), with age as a significant covariate (*p* = 0.001).

To determine specific group differences for the two strategies when adjusting for age, we conducted post-hoc covariate analyses, comparing each group against each other for the two scales. For problem-oriented action, we found significant differences between the HC group and all three clinical groups (HC vs. MD-Only: *p* < 0.001, HC vs. AN-Only: *p* < 0.001, HC vs. MD+AN: *p* < 0.001) when adjusting for age. We also found significant differences between the MD-Only and the AN-Only groups (*p* < 0.001) and the AN-Only and MD+AN groups (*p* = 0.003) when adjusting for age. MD-Only and MD+AN did not differ significantly when adjusting for age (*p* > 0.05). All significant correlations survived Bonferroni-Holm correction for multiple testing. For cognitive problem solving, we also found differences between the HC group and all clinical groups (HC vs. MD-Only: *p* < 0.001, HC vs. AN-Only: *p* < 0.001, HC vs. MD+AN: *p* < 0.001) when adjusting for age. The other groups did not significantly differ from one another when adjusting for age (all *p*_s_ > 0.05). Significant correlations again survived Bonferroni-Holm correction. Taken together, when adjusting for age in the analyses involving problem-oriented action and cognitive problem solving, exactly the same pattern of results was revealed as reported in the analyses without age as a covariate.

### Correlations Between Total Adaptive and Maladaptive ER Strategies and Symptom Level

For total adaptive ER strategies, a significant correlation was only found in the HC group, in which a higher depressive symptom level was related to the use of less adaptive strategies (*r* (81) = − 0.44, *R*^2^ = 0.19, *p* < 0.001). For total maladaptive strategies, significant correlations with depressive symptom level were found in the HC group (*r* (81) = 0.36, *R*^2^ = 0.13, *p* < 0.001), the MD-Only group (*r* (82) = 0.30, *R*^2^ = 0.09, *p* = 0.005) and the MD+AN group (*r* (23) = 0.60, *R*^2^ = 0.36, *p* = 0.002), with higher depressive symptom levels being associated with the use of more maladaptive strategies. Within the AN-Only and MD+AN groups, correlations between total adaptive and maladaptive ER strategies and eating disorder symptom level were assessed. In both groups, there were no significant correlations between total EDI-2 score and adaptive strategies (*p*_*s*_ > 0.05) or either of the three EDI-2 scales “drive for thinness”, “body dissatisfaction”, and “bulimic symptoms” (*p*_*s*_ > 0.05). Maladaptive strategies correlated with total EDI-2 score in both the AN-Only (*r* (35) = 0.35, *R*^2^ = 0.12, *p* = 0.033) and MD+AN group (*r* (21) = 0.57, *R*^2^ = 0.32, *p* = 0.005), but no correlations were significant between the three mentioned EDI-2 scales and maladaptive strategies in either the AN-Only or MD+AN group (*p*_*s*_ > 0.05). All significant correlations survived the Bonferroni-Holm procedure. Supplementary Table 4 gives an overview of the correlations between symptom level (BDI-II total score and EDI-2 total score) and total adaptive and maladaptive ER within the four groups. Supplementary Table 5 details specific correlations between each EDI-2 subscale and total adaptive and maladaptive ER within the AN Only and MD+AN groups.

## Discussion

This study investigated differences in ER between adolescent girls with diagnoses of MD, AN, and comorbid MD+AN compared to healthy controls. Our findings highlight that girls in all of the three clinical groups show deficits in ER, using more maladaptive ER strategies and less adaptive ER strategies than healthy controls. With regard to the specific ER strategies, all clinical groups reported more frequent use of the strategies giving up, withdrawal and self-devaluation and less of the adaptive strategies cognitive problem solving, problem-oriented action, forgetting, positive mood enhancement and distraction. Differences between the three clinical groups were most apparent in maladaptive strategies. The two groups comprising patients with MD (MD-Only and MD+AN) reported more maladaptive strategies than the girls with only AN, while the MD-Only and MD+AN showed no differences. This pattern could also be found in most primary strategies, indicating that difficulties in ER could be more specific to MD than AN psychopathology. Finally, maladaptive strategies were correlated with depressive psychopathology MD and MD+AN groups.

### Differences in Total Adaptive and Maladaptive ER Strategies

Our finding that all clinical groups reported more total maladaptive and less total adaptive strategies is well in line with previous findings (e.g. [17, 36]). Interestingly, we found that both the MD-Only and the MD+AN group reported more maladaptive strategies than the AN-Only group, with no difference between MD-Only and MD+AN. Rather than seeing a uniform exacerbation of deficits in comorbid MD+AN, it seems that compared to only AN, an additional diagnosis of MD led to a higher use of maladaptive ER, while compared to only MD, an additional diagnosis of AN did not correspond to such a higher use.

### Differences in Primary ER Strategies

Our finding that adolescents with MD did not differ from healthy adolescents in their use of revaluation is surprising, as it is well documented that adolescents with MD often show deficits in reappraisal compared to healthy youths (e.g. [63, 64]). However, while the concepts of revaluation and reappraisal are similar, there are some key differences. The FEEL-KJ measures revaluation via two items that are repeated in the context of three emotions, which mostly focus on dampening the emotional significance of the situation (“I tell myself the problem is not so bad”, “I tell myself it’s not important”), not on actually reinterpreting the situation, which is how reappraisal is often represented in the literature. This key difference could explain our divergent findings and implicates that perhaps revaluation is something adolescents with MD are capable of, but reappraisal might indeed be one aspect of ER in which these patients show deficits. Additionally, we only investigated the reported use of a certain strategy in daily life, not how efficient the adolescents are at using it. It is possible that youth with MD use reappraisal habitually, but might be inefficient in actually regulating their emotions through this strategy.

In AN, we expected a more frequent use of withdrawal and rumination and a less frequent use of acceptance, but this proved correct only for withdrawal. Acceptance was the only primary adaptive strategy that girls in the AN-Only group used just as much as healthy girls, contradicting previous findings of deficits in this specific strategy in adults with AN [13, 40]. However, as there were no previous findings of reduced use of acceptance in adolescents with AN, it is possible that this deficit is of relevance in adulthood, but not in youth. Our finding of deficits in revaluation in AN-Only is in line with findings showing deficits in adolescent and young adult patients with eating disorders compared to healthy participants from a study [39]. In addition to this an fMRI study showed reduced activation in the dorsolateral prefrontal cortex during reappraisal in adults with AN compared to healthy controls [65]. However, considering the lack of studies in adolescents and the differences between the concepts of revaluation and reappraisal, further studies are necessary to determine the scope of possible deficits in adolescents with AN.

All in all, AN-Only seems to show more frequent use of several adaptive (acceptance, problem-oriented action, positive mood enhancement) and less frequent use of several maladaptive (giving up, withdrawal) strategies than MD-Only, with the comorbid group often falling between the two groups. A possible explanation for why the AN-Only group shows less ER deficits than the MD-Only group could be that patients with AN have too much control over their emotions rather than having difficulties controlling them. This also fits with some of our findings of girls with AN-Only showing increased withdrawal and self-devaluation, as both isolating oneself and a tendency to invalidate own positive aspects can be part of this overcontrolled nature [43, 44].

Our results provide various interesting insights with respect to the ER strategies in which the MD+AN group differed from either MD-Only or AN-Only. For adaptive ER, girls with AN-Only displayed more problem-oriented action than girls with MD+AN or MD-Only. It is possible that deficits in problem-oriented action are specifically related to depressive psychopathology as it is one of the more active ER strategies A recent study by Kenny et al. [66] found that while symptoms of irritability and depressed mood are often shared by adolescents with AN and MD, low energy seems to be more specific for MD. Deficient problem-oriented action could therefore be at least partially attributed to low energy when MD is present. Intact problem-oriented action in girls with AN could also be due to the fact that patients with AN tend to be very perfectionistic (for a meta-analysis, see [67]). However, it should be noted that in our sample, girls with AN did not show more problem-oriented action than the HC group, they merely did not show any deficits in this strategy compared to healthy girls.

For maladaptive ER, giving up, withdrawal and self-devaluation seem to be more impacted in MD+AN than AN-Only, while deficits in rumination are more similar across the groups. Giving up, withdrawal and self-devaluation are closely related to core descriptors of MD [68]. While rumination has been extensively studied in the context of MD, there are some studies indicating that it could be a transdiagnostic construct that is also relevant to other psychopathologies including eating disorders [26, 69].

Taken together, our result pattern suggests that deficits in ER play a more important role in MD than in AN. Indeed, ER has been previously discussed as an important factor in the etiology of MD, with studies and models showing ER deficits as important precursors of MD [27, 70, 71]. In models of AN, ER deficits are currently not being considered as a precursor of the disorder in youth (e.g. [72, 73]). Thus, while our findings confirm that ER deficits are related to both MD and AN, it seems to be specifically relevant in MD and, in turn, in comorbid MD+AN.

### Correlations Between Total Adaptive and Maladaptive ER Strategies and Symptom Level

As expected, maladaptive strategies correlated with depressive symptoms in the MD-Only and MD+AN groups (e.g. [32]); however, there was no correlation between depressive symptoms and adaptive ER. Taken together, this indicates that the relationship between depressive symptomatology and deficits in ER is stronger for maladaptive strategies than for adaptive ones [26]. Aldao and Nolen-Hoeksema [26] offered a possible explanation, that the effectivity of adaptive ER strategies might be dependent on context, i.e. adaptive strategies could be employed less frequently because reappraisal is not always possible (see also [74]). Maladaptive strategies, meanwhile, are largely independent from the context of the situation and thus might exert negative effects in everyday life more often, resulting in a stronger connection to psychopathology.

Higher eating disorder psychopathology as measured by the total EDI-2 score correlated with higher use of maladaptive strategies in both AN-Only and the MD+AN group. However, when these analyses were restricted to the three EDI-2 scales particularly relevant to disordered eating behavior (drive for thinness, body dissatisfaction, and bulimic symptoms), no such relationship could be found. Interestingly, a study by Racine and Wildes [42] found that BMI was not related to emotion dysregulation over time, while conversely, depressive symptoms were a predictor for changes in ER. Taken together, this could speak to the fact that core symptoms of AN are not as strongly related to ER as depressive symptoms or other transdiagnostic factors within patients with AN.

## Limitations

Some limitations should be noted. First, our data is based exclusively on self-report measures. On one hand, this might be criticized, as the assessment of ER via self-report can be hindered by difficulties in recognizing and talking about emotional experiences, which is especially relevant in patients with AN and MD [8, 75]. On the other, the validity of self-report measures has previously been demonstrated by studies finding a substantial correlation between self-reported ER data and neural and physiological measures of ER (e.g. [76]). Additionally, we only collected cross-sectional data, so we can draw no conclusions as to potential causal influences, and we did not collect data on length of illness for girls with MD and/or AN, both of which should be subject to future studies. Finally, diagnoses of comorbid anxiety disorders were frequent in our sample, especially among the MD-Only and the comorbid group. There is evidence suggesting that the diagnosis of comorbid anxiety can be associated with habitual ER deficits in MD and AN [17, 31] and the selection and effectiveness of ER strategies itself [77]. However, as comorbidities between MD and anxiety, as well as between AN and anxiety, are high [2, 50], it could be argued that our findings approximate clinical reality more so than a ‘pure’ sample without comorbid anxiety disorders would.

## Clinical Implications

Our findings hold several important clinical implications, one of them being the importance of ER assessment in the diagnostic process for both MD and AN. In addition to providing insights into specific deficits, this could inform treatment decisions and help individualizing and possibly improving treatments effects for patients with MD and/or AN.

Based on our findings on specific deficits associated with both disorders, a focus on ER could prove beneficial in interventions for MD and/or AN. Cognitive behavior therapy has been recommended by NICE guidelines for the treatment of MD and AN in adolescence [78, 79] and is well suited to promote adaptive ER strategies and direct focus away from maladaptive coping points. As an add-on to CBT, ER training has shown beneficial effects in adults with MD [80] and similar approaches for ER training in adolescent MD are being developed [81].

Other treatments such as self-compassion training [82] and emotional acceptance therapy [83] have shown some positive effects in relieving eating disorder pathology in adults with AN. RO DBT has also been proposed as a treatment option for AN. RO DBT focuses on reducing maladaptive behaviors associated with overcontrol, such as emotional inhibition and behavioral avoidance, and emphasizes building skills to increase openness, flexibility, and social connection. Studies have shown positive effects for RO-DBT in adults [43, 44] with AN and there are first preliminary results from a case series revealing beneficial results in adolescents as well [84]. Together, these approaches show that targeting maladaptive behaviors such as inhibition and tendencies to self-devaluate and withdraw from emotions and can be beneficial.

## Conclusions

In conclusion, this study examined differences in habitual ER between adolescent girls with MD, AN, their comorbid presentation and healthy girls. Our findings highlight that both MD and AN are marked by difficulties in ER, specifically a more frequent use of maladaptive ER strategies and a less frequent use of adaptive ones. As the first study to compare the ER profiles of comorbid MD and AN with their singular presentations, we found that compared to only AN, an additional diagnosis of MD is related to a more frequent use maladaptive ER, while compared to only MD, an additional diagnosis of AN is not related to more frequent use. When looking at specific ER strategies, a comorbid diagnosis seems to be associated with deficits in similar areas as only MD and only AN, but not with a universal exacerbation of these deficits.

## Summary

Major depression (MD) and anorexia nervosa (AN) are prevalent disorders in adolescence with high rates of comorbidity. Both are characterized by deficits in emotion regulation (ER), however, studies that have directly compared ER profiles between patients with MD and AN are rare. Moreover, it is largely unexplored whether patients suffering from both conditions show additive deficits in ER. This study examined the habitual use of adaptive and maladaptive ER strategies in 229 adolescent girls, aged 12–18 years, with MD-Only (n = 84), AN-Only (n = 37), comorbid MD+AN (n = 25), and healthy girls (n = 83). We also investigated relationships between the severity of depressive and eating disorder symptoms and the use of adaptive and maladaptive ER. Girls with MD, AN and comorbid MD+AN all habitually reported more maladaptive and less adaptive ER strategies than healthy girls. MD-Only and MD+AN groups showed more frequent use of maladaptive ER compared to the group with only AN, with no differences between only MD and MD+AN. In the MD-Only and MD+AN groups, depressive symptoms were positively correlated with maladaptive ER. The results suggest that comorbid MD+AN is not necessarily associated with a uniform addition of ER deficits, rather, an additional diagnosis of MD exacerbates impairments in AN. Identifying specific ER profiles can provide important targets in prevention and treatment for AN, MD and MD+AN. The relationship between psychopathology and ER seems to be stronger for maladaptive than adaptive ER. Identifying specific profiles in ER deficits can provide important targets in prevention and treatment for AN, MD and their comorbid presentation.

## Supplementary Information

Below is the link to the electronic supplementary material.

Supplementary file1 (DOCX 37 kb)
