# Supplementary material for: Emotion Regulation Deficits in Adolescent Girls with Major Depression, Anorexia Nervosa and Comorbid Major Depression and Anorexia Nervosa
Source: Child Psychiatry Hum Dev. 2022 Apr 12;54(5):1476–88. doi: 10.1007/s10578-022-01353-4 (PMC10435395; doi:10.1007/s10578-022-01353-4)
Supplement: Supplementary file 1 — Supplementary file1 (DOCX 37 kb) [file 10578_2022_1353_MOESM1_ESM.docx]

# Supplementary Tables

Supplementary Table 1. ER Strategy Definitions

|  | | Description  Example items: “When I am angry / sad / anxious… |
| --- | --- | --- |
| Adaptive ER Strategies | | |
|  | Acceptance | Accepting the current emotional state and what caused it, making the best of the current situation.  “… I accept what makes me angry / sad / anxious” |
|  | Cognitive Problem Solving | Thinking about possible solutions to the current problem or situation or about possible courses of action.  „...I think about what I could do” |
|  | Problem-Oriented Action | Changing the current situation or actively working towards solving the problem.  „...I try to change what makes me angry / sad / anxious” |
|  | Positive Mood Enhancement | Thinking happy thoughts or remembering good memories.  „...I think about things that make me happy” |
|  | Revaluation | Lessening the emotional significance or cognitive relevance of the situation.  „...I tell myself the problem is not so bad” |
|  | Forgetting | Trying to forget the current problem or telling oneself that it will pass.  „...I try to forget what makes me angry / sad / anxious” |
|  | Distraction | Doing something fun or distracting.  „...I do something that makes me happy” |
| Maladaptive ER Strategies | | |
|  | Giving Up | Doing nothing, having the mindset that nothing can be done to solve the current problem or change your emotions about it.  „...I don’t want to do anything at all” |
|  | Aggression | Fighting or acting aggressively toward others.  „...I take my emotions out on other people” |
|  | Withdrawal | Avoiding social interaction and confrontation with the situation or one’s emotions.  „...I withdraw into myself” |
|  | Self-Devaluation | Blaming oneself for the current problem.  “…I look for mistakes I could have made” |
|  | Rumination | Repeatedly thinking about the reasons for the current problem  “…I keep thinking about why I am angry / sad / anxious” |

Supplementary Table 2. T-Values for Primary and Total Adaptive and Maladaptive FEEL-KJ Scales

|  | | HC  (n = 84) | MD Only  (n = 84) | AN Only  (n = 37) | MD+AN  (n = 25) |
| --- | --- | --- | --- | --- | --- |
|  | | *M* (*SD)* | *M* (*SD)* | *M* (*SD)* | *M* (*SD)* |
| Total Adaptive ER Strategies | | 52.23 (10.17) | 36.93 (10.41) | 41.57 (10.56) | 36.84 (7.89) |
|  | Acceptance | 50.31 (10.45) | 37.85 (10.54) | 45.11 (11.47) | 39.32 (7.54) |
|  | Cognitive Problem Solving | 53.05 (9.47) | 43.37 (12.05) | 46.76 (8.99) | 44.24 (9.31) |
|  | Problem-Oriented Action | 52.77 (11.12) | 35.74 (11.20) | 44.43 (11.12) | 36.92 (8.00) |
|  | Positive Mood Enhancement | 51.92 (9.65) | 36.69 (7.46) | 41.22 (8.40) | 38.76 (7.14) |
|  | Reappraisal | 52.76 (8.96) | 51.08 (13.25) | 46.68 (10.86) | 48.52 (12.42) |
|  | Forgetting | 49.42 (9.27) | 43.38 (10.96) | 43.62 (11.71) | 39.04 (8.23) |
|  | Distraction | 51.23 (10.72) | 35.15 (8.24) | 39.68 (9.98) | 36.28 (8.08) |
| Total Maladaptive ER Strategies | | 45.43 (10.96) | 66.87 (9.63) | 57.97 (12.22) | 66.68 (10.48) |
|  | Giving Up | 43.77 (11.09) | 67.65 (8.44) | 55.95 (11.58) | 63.40 (11.07) |
|  | Aggression | 47.00 (9.26) | 49.01 (11.30) | 50.30 (13.02) | 50.28 (10.88) |
|  | Withdrawal | 48.45 (9.19) | 65.40 (8.76) | 59.19 (8.63) | 65.04 (9.13) |
|  | Self-Devaluation | 47.40 (9.89) | 63.63 (13.26) | 57.19 (14.62) | 66.08 (10.53) |
|  | Rumination | 49.12 (10.01) | 55.56 (10.30) | 51.22 (9.55) | 55.20 (10.90) |

*Note.* HC = healthy control. MD = major depression. AN = anorexia nervosa. MD+AN = major depression and anorexia nervosa, *M* = mean; *SD* = standard deviation.

Supplementary Table 3. Internal Consistencies

| Measures | | | Cronbach’s α |
| --- | --- | --- | --- |
| BDI-II | | | .96 |
| EDI-2 | | | .97 |
| FEEL-KJ | | |  |
|  | *Total Adaptive Strategies* | | .96 |
|  |  | Acceptance | .82 |
|  |  | Cognitive Problem Solving | .87 |
|  |  | Problem-Oriented Action | .86 |
|  |  | Positive Mood Enhancement | .90 |
|  |  | Reappraisal | .85 |
|  |  | Forgetting | .77 |
|  |  | Distraction | .93 |
|  | *Total Maladaptive Strategies* | | .92 |
|  |  | Giving Up | .88 |
|  |  | Aggression | .82 |
|  |  | Withdrawal | .86 |
|  |  | Self-Devaluation | .87 |
|  |  | Rumination | .73 |

Supplementary Table 4. Correlations between Symptom Level and Total Adaptive and Maladaptive ER Strategies

|  | | HC  (n = 84) | MD Only  (n = 84) | AN Only  (n = 37) | MD+AN  (n = 25) |
| --- | --- | --- | --- | --- | --- |
| Depressive Symptoms (BDI-II) | |  |  |  |  |
|  | Total Adaptive ER Strategies | -.44** | -.10 | .12 | .01 |
|  | Total Maladaptive ER Strategies | .36** | .30* | .23 | .60** |
| Eating Disorder Symptoms (EDI-2) | |  |  |  |  |
|  | Total Adaptive ER Strategies | – | – | -.19 | -.13 |
|  | Total Maladaptive ER Strategies | – | – | .35* | .58* |

*p < .05, **p < .01

*Note:* Correlational results have been corrected for multiple comparisons with the Bonferroni-Holm correction. HC = healthy control. MD = major depression. AN = anorexia nervosa. MD+AN = major depression and anorexia nervosa, BDI = Beck’s Depression Inventory, EDI = Eating Disorder Inventory.

Supplementary Table 5. Correlations between EDI-2 Subscales and Total Adaptive and Maladaptive ER Strategies

|  | Total Adaptive ER Strategies | |  | Total Maladaptive ER Strategies | |
| --- | --- | --- | --- | --- | --- |
|  | AN Only  (n = 37) | MD+AN  (n = 25) |  | AN Only  (n = 37) | MD+AN  (n = 25) |
| Drive for Thinness | .06 | -.32 |  | .10 | -.02 |
| Bulimic Symptoms | -.05 | .19 |  | -.11 | .13 |
| Body Dissatisfaction | -.03 | -.30 |  | -.04 | .19 |
| Ineffectiveness | -.20 | -.26 |  | .37 | .60* |
| Perfectionism | .03 | -.18 |  | .37 | .54 |
| Interpersonal Distrust | -.19 | .27 |  | .35 | .20 |
| Interoceptive Awareness | -.25 | .06 |  | .29 | .47 |
| Maturity Fears | -.10 | .28 |  | .14 | .13 |
| Asceticism | -.05 | -.14 |  | .37 | .57* |
| Impulse Regulation | -.17 | .02 |  | .39 | .41 |
| Social Insecurity | -.42 | -.16 |  | .22 | .31 |

*p < .05

*Note:* Correlational results have been corrected for multiple comparisons with the Bonferroni-Holm correction. AN = anorexia nervosa. MD+AN = major depression and anorexia nervosa.
